# Cell-free synthesis of functional human epidermal growth factor receptor: Investigation of ligand-independent dimerization in *Sf*21 microsomal membranes using non-canonical amino acids

**DOI:** 10.1038/srep34048

**Published:** 2016-09-27

**Authors:** Robert B. Quast, Biljana Ballion, Marlitt Stech, Andrei Sonnabend, Balázs R. Varga, Doreen A. Wüstenhagen, Péter Kele, Stefan M. Schiller, Stefan Kubick

**Affiliations:** 1 Fraunhofer Institute for Cell Therapy and Immunology (IZI), Branch Bioanalytics and Bioprocesses (IZI-BB), Am Mühlenberg 13, D-14476 Potsdam, Germany; 2 Institute for Macromolecular Chemistry, University of Freiburg, Stefan-Meier-Str. 31, D-79104 Freiburg, Germany; 3 Freiburg Institute for Advanced Studies (FRIAS), School of Soft Matter Research, University of Freiburg, Albertstr. 19, D-79104 Freiburg, Germany; 4 Center for Biosystems Analysis (ZBSA), University of Freiburg, Habsburger Str. 49, D-79104 Freiburg, Germany; 5 Chemical Biology Research Group, Hungarian Academy of Sciences, CNS, IOC, Magyar tudósok krt. 2, H-1117 Budapest, Hungary.

## Abstract

Cell-free protein synthesis systems represent versatile tools for the synthesis and modification of human membrane proteins. In particular, eukaryotic cell-free systems provide a promising platform for their structural and functional characterization. Here, we present the cell-free synthesis of functional human epidermal growth factor receptor and its vIII deletion mutant in a microsome-containing system derived from cultured *Sf*21 cells. We provide evidence for embedment of cell-free synthesized receptors into microsomal membranes and asparagine-linked glycosylation. Using the cricket paralysis virus internal ribosome entry site and a repetitive synthesis approach enrichment of receptors inside the microsomal fractions was facilitated thereby providing analytical amounts of functional protein. Receptor tyrosine kinase activation was demonstrated by monitoring receptor phosphorylation. Furthermore, an orthogonal cell-free translation system that provides the site-directed incorporation of *p*-azido-L-phenylalanine is characterized and applied to investigate receptor dimerization in the absence of a ligand by photo-affinity cross-linking. Finally, incorporated azides are used to generate stable covalently linked receptor dimers by strain-promoted cycloaddition using a novel linker system.

Cell-free protein synthesis (CFPS) has emerged to be a versatile tool for the synthesis of difficult-to-express proteins including membrane proteins^1^. Nevertheless, functional and structural investigations of membrane proteins using conventional cell-free systems generally require the addition of supplements such as detergents, surfactants and membrane mimetics to facilitate proper protein folding^2^. Alternatively, laborious re-folding strategies become essential to obtain native and functional products^3^. A promising alternative to these conventional systems has been developed, which is based on cell extracts derived from cultured *Sf*21 cells^4^. This system contains microsomal structures originating from the ER, which provide a nature-like membranous environment that can serve as a platform for structural and functional *in vitro* investigations of properly folded and post-translationally modified membrane proteins^5^. The yields typically obtained in the *Sf*21 system are in the range of several μg per ml^6^. To improve the system’s productivity, viral internal ribosome entry sites (IRES) have been reported to increase total protein yields. In particular, the use of IRES elements that promote protein translation independent of eukaryotic initiation factors, such as the cricket paralysis virus IRES (CrPV-IRES), circumvent a major bottleneck observed in eukaryotic CFPS^7^.

Orthogonal translation systems facilitating the incorporation of non-canonical amino acids have gained much attention over the last decades, providing researchers with efficient tools to implement desired characteristics into proteins in a site-directed manner^8^,^9^,^10^,^11^. In this context, CFPS offers several advantages that arise from the direct accessibility of the protein translation process^12^. Orthogonal suppressor tRNA/synthetase pairs mediating the incorporation of non-canonical amino acids by stop codon suppression, can now be supplemented directly to the open cell-free system in a purified form, together with high concentrations of non-canonical amino acids in order to achieve optimal incorporation efficiencies. A widely used example of such a non-canonical amino acid is p-azido-L-phenylalanine (AzF), which can be used for protein interaction studies due to its photosensitivity^13^. Furthermore, AzF is known to selectively react under physiological conditions via a set of different reactions such as Staudinger ligation, copper-catalyzed alkyne-azide cycloadditions and copper-independent strain-promoted alkyne-azide cycloadditions^14^. In this context, bioorthogonal chemistry facilitates functional analysis of membrane proteins.

A prominent example of a sophisticated eukaryotic transmembrane protein is the epidermal growth factor receptor (EGFR), representing one of the best-studied human receptor tyrosine kinases. It is involved in a variety of processes that support cell viability and regulate cell-growth, differentiation and migration^15^. Correspondingly, malfunction has been connected to a variety of diseases and EGFR signaling plays a pivotal role in different types of cancer^16^. The EGFR is a high-molecular weight glycoprotein of approximately 180 kDa with a single transmembrane-spanning segment that connects its extracellular domains, including the ligand binding site to the intracellular domains, comprising a tyrosine kinase domain followed by a highly dynamic C-terminal tail. Upon activation, selected residues in the C-terminal tail are phosphorylated that serve as recognition sites for different adapter molecules thereby translating extracellular signals into the cell^17^. Dimerization is the predominant model describing signal transduction initiation of the EGFR. Interestingly, besides ligand induced dimer formation, the EGFR has also been found to dimerize in the absence of a ligand^18^. Moreover, even autophosphorylation activity has even been described to occur without bound ligand, which seems to be promoted by a high receptor density inside the membrane^19^. Until today, the mechanistic details of receptor tyrosine kinase transactivation in the absence of a ligand remain poorly understood.

In this study, we describe the CrPV-IRES-mediated cell-free synthesis of the full-length glycosylated EGFR in a microsome-containing *Sf*21 system. Furthermore, an enrichment of receptors in the *Sf*21 microsomes is achieved either by a repetitive synthesis approach or a combination of IRES-mediated synthesis together with supplementation of the reaction with poly G. The receptor’s embedment in the microsomal membranes is verified and functionality in the absence of ligand, supported by increased receptor densities inside the membranes, is assessed by detection auf receptor phosphorylation. Finally, the ability of an orthogonal cell-free translation system (OcfTS) composed of a mutant *E. coli* tyrosyl-tRNA synthetase and a natural amber suppressor tRNA_CUA_ to incorporate the non-canonical amino acid *p*-azido-L-phenylalanine (AzF) into cell-free synthesized EGFR at different positions in a site-directed manner and with high fidelity is demonstrated. Based on its inherent characteristics, the incorporated azido group is further utilized to gain evidence on receptor dimerization through photo-cross-linking. In this context, the generation of covalently linked synthetic EGFR dimers is facilitated by a novel homobifunctional COMBO linker system.

## Results

### Cell-free synthesis and characterization of functional EGFR

**Figure 1:**
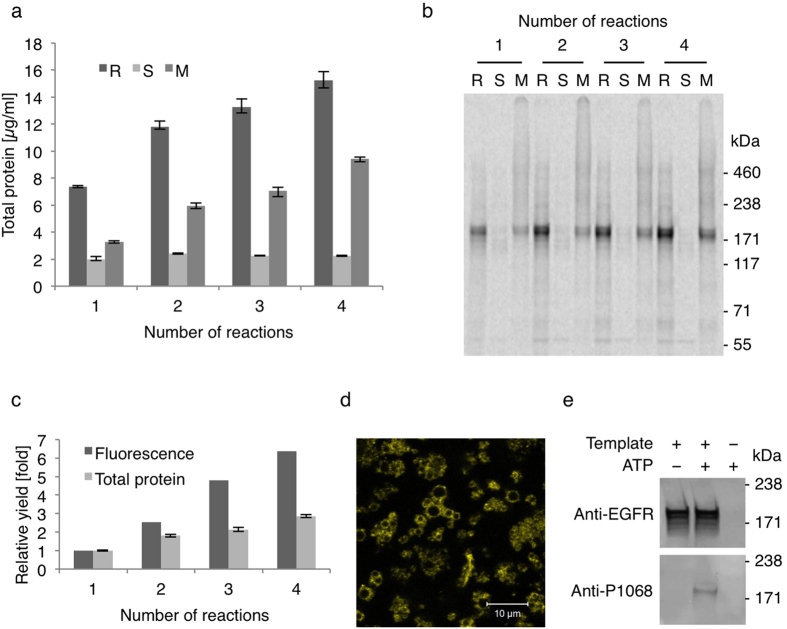
Enrichment of functional EGFR-eYFP in the *Sf*21 microsomal fraction by repetitive cell-free synthesis. (**a**) Total protein yields after each cell-free reaction in the complete reaction mixture (R), the supernatant fraction (S) and the microsomal fraction (M). Error bars represent the standard deviation of triplicate analysis. (**b**) Autoradiography of corresponding samples after electrophoretic separation under denaturing conditions. (**c**) Yields of total protein and eYFP fluorescence after each cell-free reaction relative to the first reaction. (**d**) Confocal fluorescence image of EGFR-eYFP in microsomal fraction taken under hypoosmotic buffer conditions. (**e**) Western Blot of microsomal fractions with and without EGFR-eYFP after incubation in kinase buffer. Isotopic labeling was achieved by ^14^C-leucine supplementation. The western blots (**e**) have been adapted in contrast, brightness and sharpness for better visibility. The original images can be found in Supplementary Fig. 1.

The synthesis of EGFR-eYFP based on the standard conditions previously described for the cell-free *Sf*21 system^6^ yielded relatively low amounts of several μg/ml within 90 minutes of reaction time (Fig. 1a). Although the successful synthesis of the EGFR-eYFP fusion protein was verified by different means (Fig. 1a–c), ligand-independent receptor activation based on autophosphorylation of Y1068 was not detectable (data not shown). Therefore, a batch of inherent *Sf*21 microsomes was repeatedly addressed by four consecutive cell-free reactions in order to enrich the cell-free synthesized EGFR-eYFP in the microsomal fraction and potentially promote ligand-independent receptor activation (Fig. 1a–c). As a result, an almost 3-fold increase of *de novo* synthesized EGFR was detected in the microsomal fraction yielding nearly 10 μg/ml of total protein (Fig. 1c,a, respectively). Autoradiography of isotopically labeled proteins revealed the predominant cell-free synthesis of two variants of the full-length receptor migrating at an apparent molecular weight slightly higher than the calculated 163 kDa (Fig. 1b). It should be noted that all cell-free reactions and assays presented within this study were performed in the presence of the caspase inhibitor Z-VAD-FMK to prevent degradation of cell-free synthesized EGFR, which was observed during a prolonged incubation in the reaction mixture in the absence of the inhibitor (Supplementary Fig. 2). Interestingly, the relative increase of fluorescence based on the eYFP fusion showed a higher increase with each additional cell-free reaction than was observed for the total protein (Fig. 1c). As expected, fluorescent spheres in the confocal image of the microsomal fraction taken under hypo-osmotic conditions after four consecutive cell-free reactions reflected the EGFR-eYFP fusion protein to be localized at the microsomal membranes, thereby supporting the hypothesis of membrane embedment due to a directed translocation mediated by the N-terminal melittin signal peptide in the cell-free environment (Fig. 1d). Finally, this enabled the detection of the Y1068 (corresponds to Y1090 in the synthesized construct including the melittin signal peptide) phosphorylation after incubation of the microsomal fraction in kinase buffer in the absence of ligand (Fig. 1e).

**Figure 2:**
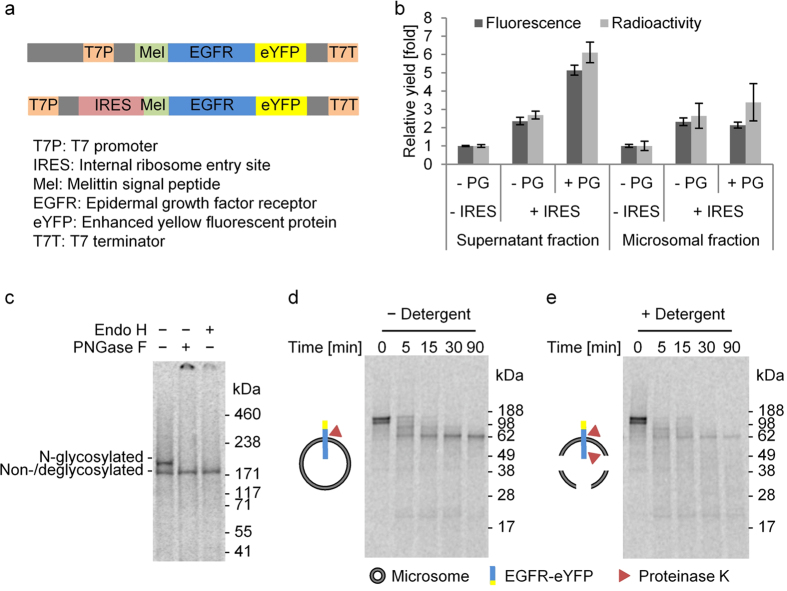
IRES-mediated cell-free synthesis of N-glycosylated EGFR-eYFP and its embedment in *Sf*21 microsomal membranes. (**a**) Schematic representation of standard (top) and IRES template (bottom) including regulatory elements. (**b**) Yields of total protein and eYFP fluorescence obtained using the standard (−IRES) or IRES template (+IRES) in the absence (−PG) or presence of poly G (+PG) relative to the standard reaction (−IRES, −PG). Error bars represent the standard deviation of triplicate analysis. (**c–e**) Autoradiography of IRES-mediated synthesis reactions in the presence of poly G after electrophoretic separation. **c**) Deglycosylation assay of complete reaction mixtures with Endo H or PNGase F. (**d,e**) Protease protection assay of microsomal fractions with Proteinase K in the absence (−Detergent) or presence of Triton X-100 (+Detergent). Isotopic labeling was achieved by ^14^C-leucine supplementation.

Although the repetitive synthesis enabled assessment of the functionality of cell-free synthesized EGFR-eYFP, the underlying methodology is comparatively laborious as well as time and material consuming. Each reaction needs to be individually constituted using fresh components followed by consecutive incubation steps. Thus, in order to increase the yield in a single batch reaction, the influence of the CrPV-IRES was investigated, which has been described to provide an enhanced synthesis rate in different eukaryotic cell-free systems due to protein translation proceeding independent of initiation factors^7^. Additionally, the supplementation with poly G was investigated, which has been reported to inhibit RNase activity in a cell-free wheat germ system^20^. The CrPV-IRES was implemented into the corresponding vector upstream of the EGFR-eYFP gene with the ATG start codon of the melittin signal sequence substituted by GCT (Fig. 2a). In this way, total protein yields were increased in the supernatant as well as in the microsomal fraction. An additional increase in protein yield was observed in the presence of poly G (Fig. 2b and Table 1, WT). The increase in total protein yield was slightly higher than the increase in fluorescence of the eYFP fusion protein. IRES-mediated synthesis in the presence of poly G provided the highest increase with 12.8 μg/ml in the supernatant fraction and 8 μg/ml in the microsomal fraction, whereas supplementation with poly G completely inhibited cell-free protein synthesis using the DNA template without IRES (data not shown). The reaction lifetime was not prolonged and remained at 90 to 120 minutes according to the reaction lifetime of the standard reaction without IRES (Supplementary Fig. 3). Protein integrity of IRES-mediated cell-free synthesized EGFR-eYFP in the presence of poly G was verified by SDS-PAGE followed by autoradiography. Treatment of reaction aliquots with PNGase F and Endo H revealed the before-mentioned two protein variants representing the N-glycosylated as well as non-glycosylated form of the receptor (Fig. 2c).

As the localization of the EGFR-eYFP fusion protein was found to be at the microsomal membranes (Fig. 1d) and protein N-glycosylation as well as proper folding and corresponding functionality would most likely require the receptors to be integrated into the microsomal membranes in a defined orientation, a protease protection assay with proteinase K was performed (Fig. 2d,e). Proteolysis of cell-free synthesized EGFR-eYFP on intact *Sf*21 microsomes resulted in a defined degradation product migrating in good agreement with the size of approximately half of the receptor fusion protein, thereby reflecting partial protection by the microsomal membranes (Fig. 2d). Disruption of the membrane integrity by addition of detergent led to almost complete protein degradation due to access of the protease to cleavage sites within the entire receptor (Fig. 2e). Therefore, it can be concluded that the cell-free synthesized EGFR-eYFP is cotranslationally translocated into the *Sf*21 microsomal membranes mediated by the N-terminal melittin signal peptide. Nevertheless, the membrane integration seemed to be incomplete, reflected by the higher increase of protein yields in the supernatant fraction compared to the microsomal fraction (Fig. 2b). Washing of the microsomes with urea revealed roughly 75% of receptor molecules to be integrated into the lipid bilayer (Supplementary Fig. 4). Although the attempt to increase the efficiency of membrane integration by supplementing higher amounts of *Sf*21 microsomes was successful and the portion of glycosylated receptor molecules increased, as a consequence the phosphorylation activity was abolished (Supplementary Fig. 5). Therefore, the amount of microsomes was left unchanged in the following experiments.

### An orthogonal cell-free translation system for AzF incorporation

We previously established an OcfTS based on the microsome-containing *Sf*21 extract and expanded by a mutant *E. coli* tyrosyl-tRNA synthetase together with a natural suppressor tRNA_CUA_^21^. Based on these results, cell-free reactions were carried out in the linked mode enabling the co-translational incorporation of AzF by amber suppression with good fidelity upon addition of appropriate mRNA templates. Unfortunately, synthesis of the wild type EGFR-eYFP from a DNA template without internal amber codon in the coupled variant of the OcfTS, were mRNA transcription and protein translation were carried out in the same reaction vessel, and subsequent treatment with a fluorescent phosphine dye, revealed selective Staudinger ligation due to misincorporation of AzF (Supplementary Fig. 6). Selected controls revealed this to arise from an insufficient specificity of the mutant synthetase AzFRS for the suppressor tRNA_CUA_. Therefore, an additional mutation (R265) was introduced into the synthetase gene according to Takimoto *et al*. in order to enhance the enzyme’s recognition of the CUA anticodon^22^. The resultant enhanced mutant synthetase eAzFRS provided an increased incorporation efficiency of AzF (Supplementary Fig. 7) but more important also facilitated a higher specificity of incorporation using standard reaction conditions (Supplementary Fig. 6).

**Figure 3:**
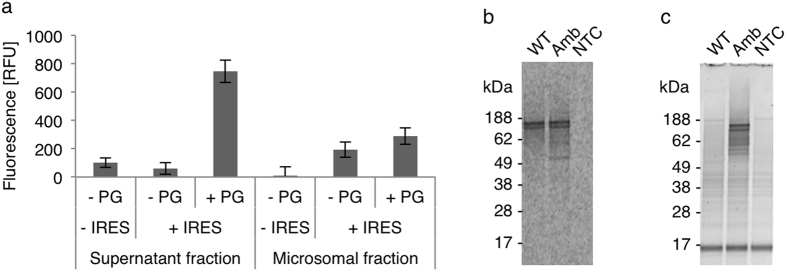
IRES-mediated cell-free synthesis of EGFR-eYFP-AzF687 by amber suppression using an orthogonal tRNA/synthetase pair and subsequent chemo-selective fluorescence modification. (**a**) eYFP fluorescence obtained using the standard template (−IRES) and the IRES template with an amber codon at position 687 (+IRES) in the absence (−PG) and the presence of poly G (+PG). Error bars represent the standard deviation of triplicate analysis. (**b**) Autoradiography and (**c**) in-gel fluorescence of microsomal fractions from reactions performed using the IRES template without (WT) and with amber codon (Amb) in the presence of poly G after treatment with DyLight650 phosphine. Isotopic labeling was achieved by ^14^C-leucine supplementation. NTC: control reaction without DNA template.

With the aim to further increase the yields of full-length suppression product, we investigated the influence of IRES-mediated synthesis and poly G in the coupled OcfTS. For this purpose, amber codons were introduced into the EGFR-eYFP templates with and without IRES, at the position corresponding to amino acid 687 in the translated receptor (corresponds to V665 in the wild type receptor without signal peptide) and synthesis of the full-length suppression product was measured based on fluorescence of the eYFP moiety only being translated upon successful amber suppression. Without additional Mg^2+^, which was previously described to enhance the productivity of the OcfTS^6^, only a small amount of suppression product was synthesized using standard conditions. The suppression product was predominantly found in the supernatant fraction (Fig. 3a, −IRES, −PG). Using the IRES template and adjusted buffer conditions^7^ with elevated levels of K^+^ and Mg^2+^, yields increased in the microsomal fraction in the absence of poly G (Fig. 3a, +IRES, −PG). According to the findings for the wild type protein, highest yields were obtained using the IRES template and supplementing the cell-free reaction with poly G (Fig. 3a, +IRES, +PG). In accordance with the results obtained for the wild type EGFR-eYFP (Fig. 2b), the increase in the supernatant fraction was higher than in the microsomal fraction (Fig. 3a). Total yields of the suppression product, estimated from corresponding yields of the wild type protein, were 4.47 μg/ml in the supernatant fraction and 2.66 μg/ml in the microsomal fraction (Table 1, Amb). The incorporation of AzF, using the adapted conditions, was verified by selective Staudinger ligation of cell-free synthesized receptors in the microsomal fraction with the fluorescent phosphine dye (Fig. 3c, Amb). The specificity of incorporation was found to be good, as no fluorescence modification was detected for the wild type control (Fig. 3c, WT).

### Ligand-independent dimerization

**Figure 4:**
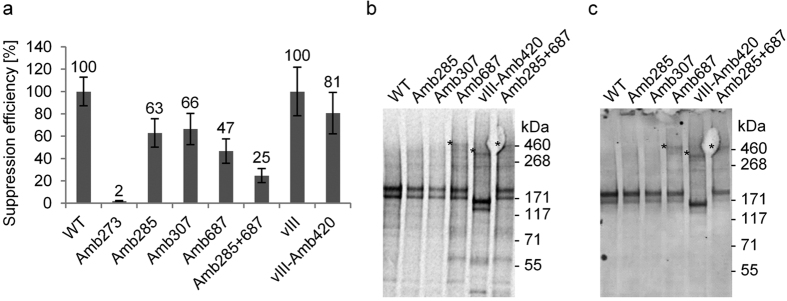
Suppression efficiency of different EGFR-eYFP amber variants and photo-affinity cross-linking of receptors in *Sf*21 microsomal membranes. (**a**) Suppression efficiency of EGFR-eYFP templates with amber codons at positions 273, 285, 307, 687, 285 + 687 and vIII-deletion mutant at position 420 based on eYFP fluorescence from complete reaction mixture in relation to wild type and vIII template without amber codon. Error bars represent the standard deviation of triplicate analysis. (**b**) Autoradiography and (**c**) western blot of phosphotyrosine 1068 of selected constructs enriched in microsomal fraction by four consecutive cell-free reactions after photo-affinity cross-linking in kinase buffer. Isotopic labeling was achieved by ^14^C-leucine supplementation. Asterisks mark the covalently cross-linked dimers. The autoradiogram (**b**) and western blot (**c**) have been adapted in contrast, brightness and sharpness for better visibility. The original images can be found in Supplementary Fig. 9.

In order to investigate ligand-independent dimerization in the microsomal membranes, a set of amber mutants was generated to introduce AzF into selected positions in the extracellular (Amb285, Amb307) and intracellular domains (Amb687) of the cell-free synthesized EGFR-eYFP. Additionally, a double mutant (Amb285 + 687) and an amber variant of the constitutively active vIII deletion mutant of the EGFR (vIII-Amb420) were generated. First, the suppression efficiency of the different amber variants in the OcfTS was analyzed in relation to the synthesis of the corresponding receptor from templates without internal amber codon (Fig. 4a and Supplementary Fig. 8). All mutants were successfully synthesized but with varying efficiencies. The suppression efficiency decreased the more distant the amber codon was located downstream of the ATG start codon in the full-length EGFR-eYFP constructs. The extracellular mutants Amb285 and Amb307 exhibited more than 60% and the intracellular mutant Amb687 roughly 50% of suppression efficiency. The combination of two internal amber codons led to a further decrease of suppression efficiency down to 25%. This efficiency is in good accordance to previously published results, since suppression of multiple amber codons in general is reported to be very inefficient^23^. The amber variant of the vIII-deletion mutant Amb420 (corresponds to Amb687 in the full-length EGFR), which lacks 267 amino acids in the extracellular domain^24^, exhibited the highest suppression efficiency above 80%.

Based on the ability to photo-activate AzF^13^ we next performed a cross-linking experiment using the microsomal fractions enriched with the different EGFR-eYFP mutants by four consecutive cell-free reactions, in order to investigate dimerization of cell-free synthesized receptors inside the microsomal membranes in the absence of ligand. The mutated sites in the extracellular region were chosen according to interacting residues found in the dimerization loop based on a crystal structure of the soluble extracellular region of EGFR with bound ligand^25^. Although all amber mutants exhibited autophosphorylation activity (Fig. 4c), no cross-linked dimers were found when AzF was incorporated in the extracellular dimerization loop (Fig. 4b,c). In contrast, a portion of cross-linked dimers was observed when AzF was incorporated at position 687, which is located in the intracellular juxtamembrane region. Additionally, the vIII deletion mutant with AzF incorporated at the position 420, which corresponds to 687 in the full-length receptor, also revealed partial cross-linking. Overall, these findings demonstrate the dimerization of cell-free synthesized EGFR and its vIII variant thereby emphasizing proper folding and functionality of the receptors inside the *Sf*21 microsomal membranes.

### Synthetic receptor dimerization

**Figure 5:**
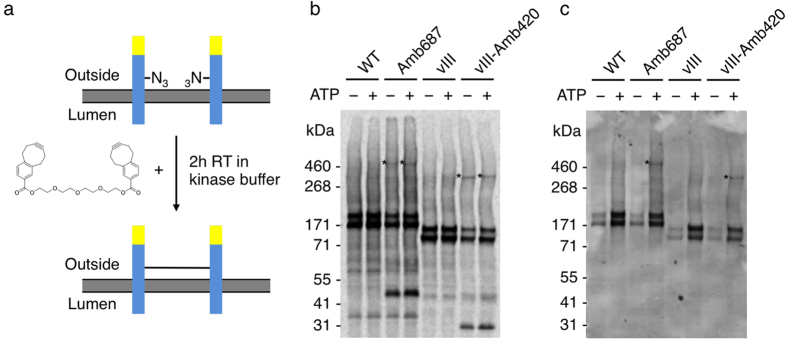
Selective cross-linking of receptors with incorporated AzF by strain-promoted cycloaddition using the bis-COMBO linker. (**a**) Schematic representation of cross-linking. Receptor- (blue) eYFP (yellow) fusion proteins are embedded in the membranes with incorporated AzF (N3) on the outside of the *Sf*21 microsomal membranes. Treatment of the microsomal fractions with the bis-COMBO linker in kinase buffer in the absence of ATP leads to strain-promoted cycloaddition to form covalently linked receptor dimers. (**b**) Autoradiography and (**c**) western blot of phosphotyrosine 1068 of selected constructs enriched in microsomal fractions by four consecutive cell-free reactions, after treatment with bis-COMBO linker. Following the cross-linking samples were incubated in kinase buffer in the absence (−) and presence of ATP (+) to allow for autophosphorylation. Isotopic labeling was achieved by ^14^C-leucine supplementation. Asterisks mark the covalently cross-linked dimers. The autoradiogram (**b**) and western blot (**c**) have been adapted in contrast, brightness and sharpness for better visibility. The original images can be found in Supplementary Fig. 10.

Finally, the two mutants that incorporate AzF in the intracellular juxtamembrane domain were used to exploit a novel cross-linking agent composed of two COMBO groups connected by a tetraethylene glycol linker in order to form stable, covalently linked receptor dimers (Fig. 5a). For that purpose, the EGFR-eYFP-AzF687 and the vIII-AzF420 mutants were enriched in the microsomal fractions by four consecutive cell-free reactions. Both proteins were subsequently treated with the bis-COMBO linker in kinase buffer in the absence of ATP. Following the cross-linking, aliquots were subjected to the tyrosine kinase assay to analyze the phosphorylation of covalently linked dimers. Autoradiography of isotopically labeled proteins revealed weak but distinct proteins migrating at approximately the molecular weight corresponding to receptor dimers (Fig. 5b). In contrast, the controls synthesized from the wild type constructs without internal amber codon were not cross-linked, underlining the specificity of the reaction between the incorporated azides and the COMBO groups. The amount of cross-linked dimers, estimated from the autoradiogram, was approximately 4% for EGFR-eYFP-AzF687 and 10% for vIII-AzF420 (Supplementary Fig. 11). Further, the phosphorylation of Y1068 was found to occur even in the pre-linked dimers, when incubated in kinase buffer in the presence of ATP (Fig. 5c).

## Discussion

In this study, we have demonstrated the cell-free synthesis of functional human EGFR in a microsome-containing system derived from cultured *Sf*21 cells that supports signal peptide mediated cotranslational translocation of the high molecular weight type I transmembrane protein. Additionally, the cell-free system is capable of equipping receptor molecules with N-glycans. Two different methodologies were investigated in order to increase the receptor concentration in the microsomal fraction, thereby promoting ligand-independent activation reflected by phosphorylation of Y1068 in an *in vitro* kinase assay. On the one hand, the standard conditions previously described for the cell-free *Sf*21 system^6^ were applied in order to enrich the receptor in the microsomal fraction by consecutive repetition of cell-free reactions performed with the same batch of *Sf*21 microsomes. On the other hand, DNA templates were equipped with the CrPV-IRES and synthesis was carried out using adapted buffer conditions and in combination with poly G. Although being comparatively laborious as well as time and material consuming, the repetitive incubation of *Sf*21 microsomes harboring cell-free synthesized EGFR-eYFP in fresh reaction mixture revealed a positive effect on protein folding reflected by the substantial increase in eYFP fluorescence compared to the increase in total protein yields in the microsomal fraction. The established protein synthesis technology enabled assessment of the functionality of cell-free synthesized EGFR-eYFP based on tyrosine phosphorylation. In comparison to the standard reaction, IRES-mediated protein synthesis provided an increase in total protein of 2.7-fold in the absence and 3.4-fold in the presence of poly G in the microsomal fractions within 120 minutes of reaction time. A major bottleneck limiting overall yields in eukaryotic CFPS is thought to arise from the translation initiation event, in particular the availability of initiation factors^26^,^27^. Moreover, phosphorylation of eIF2-alpha has been found to occur in a stress-dependent manner in insect cells^28^, thereby preventing this eukaryotic initiation factor from being recycled to participate in further initiation events. Thus, the extract preparation procedure may have an additional impact on the phosphorylation status of eIF2-alpha. Therefore, the positive effect of the CrPV-IRES on protein yields can be explained by its underlying mechanism to provide translation initiation independent of host initiation factors^7^. Additionally, eIF2-alpha phosphorylation has been connected to the induction of caspases^28^, which underlines the benefit of supplementing cell-free reactions with the caspase inhibitor Z-VAD-FMK to prevent newly synthesized proteins from degradation. Interestingly, the influence of poly G, which was previously described to improve yields in a cell-free wheat germ system by inhibiting RNases^20^, only enhanced protein yields in the context of IRES-mediated synthesis, but impaired protein production when the standard conditions were applied. Therefore, we assume that inhibition of RNase activity does not seem to be the limiting factor in our system. In contrast, poly G may directly influence the IRES-mediated translation process. The underlying mechanism however still needs to be exploited.

Overall, it can be concluded that our approach increases the rate of cell-free protein synthesis while the reaction lifetime remains unchanged. We amongst others have previously demonstrated that the reaction lifetime of the presented cell-free *Sf*21 system can be prolonged by performing reactions in a two compartment dialysis system^29^,^30^. This so called continuous exchange cell-free approach benefits from a feeding compartment, which is separated from the reaction compartment and allows for free diffusion of byproducts as well as energy equivalents and building blocks through a semipermeable membrane. In this way, membrane protein yields of more than 100 μg/ml have been reported using DNA templates lacking the CrPV-IRES used in this study. Although this approach increases the overall costs as well as time effort, it represents a valuable step towards the preparative scale production of target proteins thereby complementing our batch-based approach.

The observation that the increase of total protein was more pronounced in the supernatant fraction than in the microsomal fraction indicated a partial integration of receptor molecules into the Sf21 microsomal membranes. Treatment of the microsomes with urea further verified that in total approximately 75% of the cell-free synthesized receptors were membrane-embedded. This can be explained by an insufficient number of translocons to account for the increased protein translation rate observed when combining the IRES-mediated synthesis with the supplementation of poly G. In accordance, an increased amount of membrane-integrated receptors was observed when the amount of microsomes was increased in the cell-free synthesis reaction and likewise this provided more receptor molecules with attached glycans. In this context, it was also found that supplementation of higher amounts of Sf21 microsomes (250% and 500% in comparison to 100%) did not show any negative effects on total protein yields of EGFR-eYFP in the microsomal fraction. Nevertheless, increasing the amount of microsomes abolished the ligand independent activation of the EGFR, being in accordance with earlier findings that suggested a supportive effect of high receptor densities on its activation in the absence of ligand^19^. These results clearly demonstrate that the IRES-mediated cell-free synthesis represents a fast and economical method to produce satisfying amounts of functional EGFR for analytical purposes and the underlying cell-free system is highly tunable to meet desired requirements.

We further expanded the cell-free *Sf*21 system by an orthogonal tRNA/synthetase pair that provides the site-directed incorporation of AzF by amber suppression with high fidelity. Interestingly, we found the Arg265 substitution in the mutant synthetase to not only increase the efficiency of AzF incorporation^22^, but also improve the specificity of incorporation in our cell-free *Sf*21 system. In accordance to the before-mentioned methodologies applied for wild type EGFR synthesis, the yields of full-length suppression product obtained in the resultant OcfTS were increased by utilization of DNA templates harboring the CrPV-IRES under adapted synthesis conditions and supplementation of poly G. The investigation of different amber variants of the EGFR and the vIII deletion mutant revealed suppression efficiencies between 50% and 80% for single amber mutants and 25% for a mutant containing two internal amber codons. Compared to the efficiencies that can be obtained in cultured cells our cell-free system provides remarkable results without the need for further modifications targeted at release factors^31^ or the use of orthogonal ribosomes^23^.

Implicated by the assessment of phosphorylation and N-glycosylation in the cell-free microsome-containing environment, we were able to verify the embedment of EGFR-eYFP in the *Sf*21 microsomal membranes, most likely by active translocation mediated through the N-terminal melittin signal peptide. As ligand-independent activation was observed in the *in vitro* tyrosine kinase assay, we aimed at applying the novel OcfTS in order to investigate the dimerization of cell-free synthesized EGFR as well as the vIII deletion mutant. For that purpose, we enriched the transmembrane spanning EGFR amber variants in the microsomal fractions by four consecutive IRES-mediated synthesis reactions in the presence of poly G. Photo-affinity cross-linking revealed interaction of cell-free synthesized EGFR in the intracellular domains but not in the extracellular dimerization loop. This is in accordance with the proposed models of intramolecular tethers in the extracellular domain of the unliganded EGFR^18^. Therefore, we conclude that even though no direct contacts are present between the extracellular dimerization loops of adjacent receptors, a portion of the receptors interact in their intracellular regions, most likely in accordance with the model of an asymmetric dimer, where the acceptor juxtamembrane domain is in direct contact with the donor C-lobe of the kinase domain^32^. Moreover, we detected interaction of AzF420 in the intracellular region of the vIII deletion mutant, thereby promoting the findings of others, suggesting that asymmetric dimer formation seems to play a role in activation of the vIII mutant^33^. Finally, we generated stable covalently linked dimers of the EGFR-eYFP-AzF687 and vIII-eYFP-Amb420 using a novel bis-COMBO linker. We found phosphorylation to occur in the cross-linked dimers, indicating that the tetraethylene glycol linker provides enough freedom for the C-terminal tails to serve as substrates for the receptor tyrosine kinases. Nevertheless, if the phosphorylation of Y1068 in the covalent dimers is provided by their corresponding kinases or arises from the non-cross-linked receptors that are also present in the microsomal membranes still remains to be elucidated.

In conclusion, the presented synthesis approach represents a valuable platform for the analytical scale production of functional membrane proteins within a short period of time. Furthermore, the resulting functionalized *Sf*21 microsomes enable the functional and structural characterization of membrane-embedded proteins *in vitro* and due to the direct accessibility of the *de novo* synthesized proteins, novel methodological approaches can be employed that may be difficult or even impossible when working in cell-based systems. In this context, the utilization of non-canonical amino acids, in particular the ones that can selectively react via bioorthogonal chemistries such as the azide used in this study, bears a tremendous potential for elucidating structure-function relationships for instance using methods such as foerster resonance energy transfer. Thereby the amount of protein necessary can be significantly reduced and studies can be performed in a more complex and thus more native environment rather than on highly concentrated and purified proteins.

## Methods

### Template generation and site-directed mutagenesis

Generation of templates harboring the human EGFR gene with its native signal sequence substituted by the melittin signal sequence (Mel) and fused to eYFP (pIX3.0-Mel-EGFR-eYFP) as well as generation of the mutant *E. coli* tyrosyl-tRNA synthetase genes AzFRS (Thr37, Ser182, Ala183; pXAzFRS-SII) and eAzFRS (Thr37, Ser182, Ala183, Arg265; pQE2-eAzFRS-SII) both followed by a StrepTag II (SII; IBA) has been described previously^6^. The plasmid pIX4.0-Luc-His-Amb14 was purchased from RiNA (RiNA GmbH, Berlin, Germany). All plasmids used in this work harbor the T7 promoter and terminator sequences upstream and downstream of the ORFs, respectively. To introduce the CrPV-IRES the corresponding region was excised from a pIX3.0-CrPV-Mel-eYFP vector using NotI and BstZ17. The pIX3.0-Mel-EGFR-eYFP was cut likewise and dephosphorylated using CIP (calf intestinal alkaline phosphatase, NEB) to prevent religation of the linearized plasmid. Finally, the CrPV insert was ligated into the linearized vector pIX3.0-Mel-EGFR-eYFP to obtain pIX3.0-CrPV-EGFR-eYFP. Amber stop codons were introduced into the EGFR gene by PCR using mismatch primer pairs. The integrity of all generated constructs was verified by sequencing. Plasmid preparations suitable for cell-free protein synthesis were performed using the JETSTAR Plasmid Purification Kit (GENOMED) starting from transformed *E. coli* XL10-Gold ultracompetent cells (Agilent).

### Cell-free protein synthesis and incorporation of AzF

Preparation of the microsome-containing *Sf*21 extract as well as coupled cell-free protein synthesis using standard conditions have been described previously^6^,^7^. In brief, cell-free reactions from templates without CrPV-IRES were constituted using three different stable premixes stored at −80 °C. Premix A (10x) was composed of 300 mM HEPES-KOH (pH 7.6), 750 mM KOAc, 2.5 mM spermidine, 1 mM of the 20 standard amino acids each (Merck) and 29 mM Mg(OAc)_2_. Premix B (2.5x) contained the S7 nuclease-treated *Sf*21 extract supplemented with 250 μg/ml creatine kinase (Roche) and 50 μg/ml bulk yeast tRNA (Roche). Premix C (5x) consisted of 100 mM creatine phosphate, 8.75 mM ATP, 1.5 mM CTP, 1.5 mM UTP, 1.5 mM GTP (Roche) and 1.65 mM G(ppp)G cap analogue (Prof. Edward Darzynkiewicz, Warsaw University, Poland). First, the volume of additional RNase-free water necessary to sum up to the final reaction volume of 50 μl was calculated and pipetted into a 1.5 ml reaction tube. Next, 5 μl of premix A and 20 μl of premix B were added. Then, the caspase inhibitor Z-VAD-FMK (benzyloxycarbonyl-Val-Ala-Asp(OMe)-fluoromethylketone, Promega) was supplemented at a final concentration of 30 μM and T7 RNA polymerase (Agilent) was added to a final concentration of 1 U/μl. Finally, 10 μl premix C was added and the protein synthesis reaction was initiated by addition of template DNA at a final concentration of 60 ng/μl. Isotopic labeling was achieved by supplementation of ^14^C-leucine at 20–60 μM (specific radioactivity 25–65 dpm/pmol, Perkin Elmer). It should be noted that all components and premixes were thawed on ice, gently mixed and then stored on ice during the time of constituting the cell-free reactions. Moreover, after addition of each component, the reaction solution was gently mixed by slowly pipetting up and down and after addition of the template DNA the reaction mixture was gently mixed and spun down at 800 × g. Incubation was carried out at 27 °C and gentle shaking for 90–120 minutes (Thermomixer comfort, Eppendorf). Cell-free reactions from templates harboring the CrPV-IRES were constituted likewise but with a slightly different composition of premix A containing 2250 mM KOAc and 39 mM Mg(OAc)_2_. Poly G (30 base pair primer, IBA) was added at a final concentration of 10–15 μM by substituting the remaining RNase-free water (for comparison of total protein yields 14 μM were applied).

Enrichment of the EGFR-eYFP was achieved by repeated synthesis using the same batch of *Sf*21 microsomes. Therefore, the microsomal fraction was collected by centrifugation at 16,000 × g and 4 °C for 10 minutes after the first synthesis and resuspended in fresh reaction mixtures using the supernatant of premix B after a likewise centrifugation step. This procedure was repeated 3 times to result in four consecutive cell-free reactions.

The preparation of the mutant tyrosyl-tRNA synthetases was carried out as previously described^6^,^21^. The *in vitro* transcribed suppressor tRNA_CUA_ was purchased from RiNA (RiNA GmbH). Site-directed incorporation of AzF was achieved by supplementation of cell-free reactions with 2 μM eAzFRS, 2 μM tRNA_CUA_ and 2 mM AzF (Bachem). Incubation was performed in the dark as described above.

### Determination of total protein yields

Following cell-free protein synthesis in the presence of ^14^C-leucine, reactions were fractionated into the supernatant and the microsomal fraction by centrifugation at 16,000 × g and 4 °C for 10 minutes. Microsomal fractions were resuspended in equal volumes of PBS. Subsequently, aliquots of 5 μl (from templates without IRES) and 2.5 μl (from templates with IRES) were subjected to hot trichloroacetic acid precipitation and liquid scintillation counting. Analysis was performed in triplicates as described previously^6^. Total protein yields were calculated from measured disintegrations per minute (dpm) taking into account the specific radioactivity, the molecular mass and the number of leucines of the synthesized proteins.

### Fluorescence detection of eYFP fusion proteins

The fluorescence of the eYFP fusion proteins was measured from 5 μl aliquots of the corresponding fractions in 95 μl PBS solution on 96 well microplates (Berthold) using the “Mithras^2^ LB 943 Monochromator Multimode Reader” (Berthold). Samples were excited at 485 nm and emission was detected at 530 nm. The background fluorescence of control measurements from cell-free reactions carried out under identical conditions but in the absence of a gene template was subtracted from the measured intensities. Confocal images of the microsomal fractions were taken under hypoosmotic conditions on a LSM 510 meta (Zeiss) laser scanning microscope as described previously^6^.

### Tyrosine kinase assay

To allow for *in vitro* autophosphorylation of receptors embedded in the *Sf*21 microsomal membranes, microsomal fractions from 10 μl of the complete reaction mixture were collected and resuspended in 20 μl kinase buffer composed of 100 mM HEPES (pH 7.4), 1% glycerol, 0.1 mg/ml BSA, 5 mM MgCl_2_, 1.25 mM MnCl_2_, 0.1 mM Na_3_VO_4_, 2 μM caspase inhibitor Z-VAD-FMK and 200 μM ATP. Corresponding control reactions were performed using RNase-free water instead of ATP. Incubation was carried out for 30 minutes at room temperature.

### Endoglycosidase and Proteinase K digestion

Endoglycosidase digestion with PNGase F and Endo H (NEB) was performed on 5 μl aliquots of the complete reaction mixtures according to the manufacturer’s instructions, followed by denaturing PAGE and autoradiography as described below. The protease protection assay using Proteinase K was performed in a total volume of 10 μl, containing 5 μl aliquots of the microsomal fraction resuspended in PBS, 10 ng/μl Proteinase K (Promega), 10 mM CaCl_2_ and either 1% Triton X-100 or PBS. Incubation was carried out for 0, 5, 15, 30, 90 minutes on ice and reactions were subsequently quenched with 6.25 mM phenylmethylsulfonyl fluoride (PMSF) followed by immediate acetone precipitation.

### Photo-affinity cross-linking

To analyze the interaction of cell-free synthesized receptors inside the *Sf*21 microsomal membranes AzF was incorporated at different positions using the different templates with internal amber stop codons. The mutant proteins were enriched by four consecutive cell-free reactions and subsequently the microsomal fractions (25 μl) were resuspended in 50 μl kinase buffer with ATP and transferred to a white “Nunc F96 MicroWell Polystyrene Plate”. Photo-cross-linking was achieved by incubation on ice for 2 hours under a UV handlamp at 365 nm followed by acetone precipitation, denaturing PAGE, immunoblotting and autoradiography.

### Synthesis of bis-COMBO-TEG linker

All starting materials used in this work were obtained from commercial suppliers (Sigma-Aldrich, Acros Organics, abcr, Alfa Aesar, Merck) and used without further purification. A reaction scheme is given in Supplementary Fig. 12.

### Chromatography

For reaction progress and purity control TLC was performed on TLC silica gel 60 F254 aluminium sheets 20 × 20 cm from Merck. Visualisation was performed with a 254 nm UV lamp (low pressure Hg-Lamp from DESAGA) or by using an aqueous solution of KMnO_4_ (1.0 g KMnO_4_, 6.6 g K_2_CO_3_, 0.2 g NaOH in 100 ml water). The purification of the products was carried out by column chromatography with Geduran^®^ Si 60 Silicagel (40–63 μm) from Merck.

### NMR-Spectroscopy

^1^H- and ^13^C-NMR-Spectra were recorded on a Bruker ARX 300 spectrometer. The solvent was used as reference signal. Chemical shifts (δ) were given in parts per million (ppm) whereas the coupling constants (*J*) were reported in Hertz (Hz). Splitting patterns were designated as singlet (s), doublet (d), triplet (t), multiplet (m), doublet of doublets (dd) and broad singlet (bs).

All reactions were performed under inert gas (N_2_).

### 1,2,5,6-Tetrabromocyclooctane (1)

A solution of 1,5-*cis*-cyclooctadiene (23.76 g, 0.22 mol) and CH_2_Cl_2_ (250 ml) was cooled to −78 °C. Br_2_ (81.12 g, 1.02 mol) in CH_2_Cl_2_ (200 ml) was slowly added to the cooled solution^34^. After the addition was completed the reaction mixture was warmed to room temperature and stirred for another 1 h. A saturated aqueous solution of Na_2_S_2_O_3_ was added and stirred for a few minutes. The suspension was filtered through Celite, the two phases were separated and the aqueous phase was extracted with CH_2_Cl_2_. The combined organic layers were dried over MgSO_4_ and the solution was almost concentrated to dryness. Hexane was added to the remaining solution facilitating crystallization. The crystals were filtered off and washed with cold hexane to obtain the desired product (58.50 g, 63%) as a lightly yellow solid that includes two isomers in a ratio of 54:46.

^**1**^**H-NMR** (300 MHz, CDCl_3_): Major isomer δ = 2.33–2.49 (m, 4 H), 2.49–2.62 (m, 4 H), 4.52–4.62 (m, 4 H). Minor isomer δ = 2.10 (d, *J* = 12.4 Hz, 4 H), 2.82 (d, *J* = 13.7 Hz, 4 H), 4.76 (d, *J* = 4.2 Hz, 4 H).

^**13**^**C-NMR** (75 MHz, CDCl_3_): Major isomer δ = 31.68, 58.44. Minor isomer δ = 26.81, 57.44.

### Dibromocycloocta-1,5-diene (2)

1,2,5,6-Tetrabromocyclooctane (**1**) (58.0 g, 0.14 mol) was mixed with KO*t*Bu (38.0 g, 0.34 mol) and cooled to −78 °C. Then Et_2_O (400 ml) was added slowly to the mixture^35^. After completing the addition of Et_2_O the reaction mixture was allowed to warm up to room temperature and stirred overnight. The insoluble components were filtered off and washed with hexane. The organic phase was extracted with saturated aqueous NH_4_Cl solution dried over MgSO_4_ and purified with column chromatography using hexane as eluent. The product (30.0 g, 81%) a slightly orange oil consists of two isomers in a ratio of 63:37.

^**1**^**H-NMR** (300 MHz, CDCl_3_): Major isomer δ = 2.41 (q, *J* = 6.8 Hz, 4 H), 2.79–2.87 (m, 4 H), 6.09 (t, *J* = 7.1 Hz, 2 H). Minor isomer δ = 2.31–2.37 (m, 4 H), 2.91 (s, 4 H), 6.00–6.05 (m, 2 H).

^**13**^**C-NMR** (75 MHz, CDCl_3_): Major isomer δ = 27.56, 38.43, 124.56, 129.67. Minor isomer δ = 27.80, 38.17, 123.95, 129.99.

### Carboxymethylmonobenzobromocyclooctene (3)

Hexane (100 ml) was added to [18]-crown-6 ether (4.0 g, 0.015 mol). To this mixture first KO*t*Bu (80.0 g, 0.71 mol) and then hexane (200 ml) was added. Dibromocycloocta-1,5-diene (**2**) (30.0 g, 0.11 mol) was dissolved in hexane (200 ml) added to the reaction mixture and stirred for 1.5 h at room temperature. A saturated aqueous NH_4_Cl solution and water were added for extraction. After separation the combined organic layers were dried over MgSO_4_ and given in a clean flask. Methyl coumalate (6.0 g, 0.039 mol) was also added into the flask and stirred overnight. The solvent was evaporated and the crude product purified with column chromatography using hexane with 3% EtOAc as eluent to get a slightly orange oil (8.0 g, 25%).

^**1**^**H-NMR** (300 MHz, CDCl_3_): δ = 2.41–2.55 (m, 4 H), 2.96–3.16 (m, 8 H), 3.89 (s, 3 H), 3.90 (s, 3 H), 5.78–5.87 (m, 2 H), 7.11 (d, *J* = 7.9 Hz, 1 H), 7.18 (d, *J* = 8.5 Hz, 1 H), 7.74 (d, *J* = 1.6 Hz, 1 H), 7.77–7.85 (m, 3 H).

^**13**^**C-NMR** (75 MHz, CDCl_3_): δ = 29.62, 29.79, 32.96, 33.22, 33.27, 33.36, 39.19, 39.54, 52.11, 123.67, 124.02, 127.86, 128.07, 128.50, 128.70, 130.00, 130.23, 130.33, 130.76, 131.36, 131.79, 139.02, 139.76, 144.32, 145.08, 167.29.

### Carboxymethylmonobenzocyclooctin (COMBO) (4)

Hexane (160 ml) was added to a mixture of [18]-crown-6 ether (100 mg, 0.38 mmol) and KO*t*Bu (420 mg, 3.74 mmol). This suspension was heated to 58–60 °C then carboxymethylmonobenzobromocyclooctene (**3**) (440 mg, 1.50 mmol) was dissolved in hexane (60 ml) and added to the suspension. The reaction mixture was stirred for 90 min. After cooling to room temperature the insoluble particles were filtered off and washed with EtOAc. Ice was added. Then the two phases were separated and the organic phase was washed with water. An aqueous solution of AgNO_3_ (0.5 M) was added to the organic phase and shaken for 5 minutes. The collected aqueous phases were washed with hexane. Then a cooled ammonia solution (25%) and hexane were added. After separation the aqueous phase was again extracted with hexane. The combined organic layers were dried over MgSO_4_ and the solvent was evaporated yielding the desired product (90 mg, 29%) as colorless oil.

^**1**^**H-NMR** (300 MHz, CDCl_3_): δ = 2.20–2.39 (m, 2 H), 2.40–2.54 (m, 2 H), 2.82–2.97 (m, 2 H), 3.34–3.52 (m, 2 H), 3.90 (s, 3 H), 7.20–7.25 (m, 1 H), 7.79–7.92 (m, 2 H).

^**13**^**C-NMR** (75 MHz, CDCl_3_): δ = 22.61, 22.81, 37.61, 37.74, 52.12, 99.00, 99.41, 127.93, 128.59, 131.12, 132.03, 141.57, 146.86, 167.20.

### COMBO acid (5)

COMBO (**4**) (90 mg, 0.42 mmol) was dissolved in dioxane (7.0 ml). LiOH (200 mg, 8.35 mmol) was dissolved in water (3.0 ml) and added dropwise^36^. The reaction mixture was heated to 30 °C and stirred for 2 h. HCl (1 N) was added first and then CH_2_Cl_2_. The two phases were mixed and separated. The aqueous phase was extracted with CH_2_Cl_2_. The combined organic layers were dried over MgSO_4_. The solvent was evaporated and the residue washed with hexane to get a white solid (50 mg, 60%).

^**1**^**H-NMR** (300 MHz, 1,4-dioxane-d8): δ = 2.17–2.34 (m, 2 H), 2.34–2.48 (m, 2 H), 2.80–2.95 (m, 2 H), 3.34–3.49 (m, 2 H), 7.21–7.29 (d, *J* = 7.9 Hz, 1 H), 7.77–7.82 (dd, *J* = 1.9 Hz, *J* = 7.9 Hz, 1 H), 7.82–7.85 (d, *J* = 1.9 Hz, 1 H), 10.58 (bs, 1 H).

^**13**^**C-NMR** (75 MHz, 1,4-dioxane-d8): δ = 22.78, 22.95, 37.98, 38.16, 99.51, 100.05, 128.64, 129.53, 131.69, 132.84, 142.58, 147.67, 167.52.

### COMBO-tetraethylene glycol-COMBO (6)

CH_2_Cl_2_ (2.0 ml) was added to tetraethylene glycol (20 mg, 0.10 mmol) and cooled to 0 °C. COMBO acid (**5**) (50 mg, 0.25 mmol) was added and stirred for 15 min. N-(3-dimethylaminopropyl)-N´-ethylcarbodiimide hydrochloride (EDC) (58 mg, 0.30 mmol) and 4-dimethylaminopyridine (DMAP) (8 mg, 0.06 mmol) were dissolved in CH_2_Cl_2_ (1.0 ml) and added to the reaction mixture. The mixture was stirred for another 15 min at 0 °C then at room temperature overnight. CH_2_Cl_2_ and a saturated aqueous NH_4_Cl solution were added, mixed and the two phases were separated. The organic phase was washed with a saturated aqueous NaHCO_3_- and NaCl solution and dried over MgSO_4_. The solvent was evaporated and the crude product purified with column chromatography using CH_2_Cl_2_ with 1% MeOH as eluent to yield a colorless oil (50 mg, 36%).

^**1**^**H-NMR** (300 MHz, CDCl_3_): δ = 2.21–2.38 (m, 4 H), 2.40–2.54 (m, 4 H), 2.83–2.96 (m, 4 H), 3.46–3.52 (m, 4 H), 3.64–3.74 (m, 8 H), 3.78–3.86 (m, 4 H), 4.42–4.51 (m, 4 H), 7.22 (d, *J* = 8.5 Hz, 2 H), 7.84–7.90 (m, 4 H).

^**13**^**C-NMR** (75 MHz, CDCl_3_): δ = 22.53, 22.72, 37.47, 37.65, 64.02, 69.31, 70.72, 98.91, 99.32, 127.98, 128.45, 131.00, 132.00, 141.48, 146.88, 166.52.

### Staudinger ligation and strain-promoted cycloaddition

The modification of cell-free synthesized EGFR-eYFP with incorporated AzF at position 687 by Staudinger ligation with DyLight650 phosphine (Pierce) was carried out as previously described^6^.

The covalent cross-linking of receptor monomers with AzF incorporated in the intracellular juxtamembrane region using the bis-COMBO linker was performed on microsomal fractions enriched with receptors by four consecutive cell-free reactions. Therefore, 5 μl aliquots of the microsomal fractions were resuspended in 20 μl kinase buffer without ATP containing 10 μM of the freshly dissolved linker. Samples were incubated for 2 hour at 25 °C and subsequently centrifuged to collect the microsomes. Microsomes were then subjected to the tyrosine kinase assay in the presence or absence of ATP as described above followed by denaturing PAGE, immunoblotting and autoradiography.

### Denaturing PAGE, in-gel fluorescence, immunoblotting and autoradiography

Sample preparation including cold acetone precipitation followed by denaturing PAGE using NuPAGE 10% Bis-Tris and 3–8% Tris-Acetate precast gels (Life Technologies) as well as detection of in-gel fluorescence and autoradiography were performed as described previously and in accordance to the manufacturer’s instructions^6^. Due to increased protein concentrations resulting from utilization of the CrPV-IRES the amount of sample applied to the denaturing page was reduced to correspond to 2.5 μl of the initial fractions from the cell-free reactions.

Immunoblotting was performed using the “IBlot Gel Transfer Device” (Life Technologies) according to the manufacturer’s instructions. Following denaturing PAGE (3–8% Tris-Acetate) proteins were transferred to a PVDF membrane (Life Technologies). The membrane was blocked in “Roti-Block” (Roth) for 4 hours and subsequently incubated with “EGF Receptor (D38B1) XP^®^ Rabbit mAb 4267” or “Phospho-EGF Receptor (Tyr1068) (D7A5) XP^®^ Rabbit mAb 3777” primary antibodies diluted 1:1000 over night at 4 °C. “Anti-rabbit IgG, HRP-linked Antibody 7074” diluted 1:2000 was used as a secondary antibody and detection was carried out using the “Amersham ECL Prime Western Blotting Detection Reagent” (GE Healthcare) and the “Typhoon Trio + Variable Mode Imager” (GE Healthcare). All antibodies were purchased from “Cell Signaling Technology Inc.”. After detection, blotting membranes were dried and subjected to autoradiography.

## Additional Information

**How to cite this article**: Quast, R. B. *et al*. Cell-free synthesis of functional human epidermal growth factor receptor: Investigation of ligand-independent dimerization in *Sf* 21 microsomal membranes using non-canonical amino acids. *Sci. Rep.* **6**, 34048; doi: 10.1038/srep34048 (2016).

## Supplementary Material

Supplementary Information

## Figures and Tables

**Table 1:** Total protein yields of wild type EGFR-eYFP (WT) and full-length suppression product (Amb) obtained using the corresponding standard as well as the IRES template in the absence and presence of poly G, respectively.

Template	Fraction	IRES	Poly G	Total protein [μg/ml]
WT	Supernatant fraction	−	−	2.10 ± 0.16
		+	−	5.65 ± 0.15
		+	+	12.84 ± 0.68
	Microsomal fraction	−	−	2.37 ± 0.60
		+	−	6.28 ± 0.27
		+	+	8.04 ± 1.30
Amb	Supernatant fraction	−	−	0.51 ± 0.05^a^
		+	−	0.34 ± 0.04^a^
		+	+	4.47 ± 0.40^a^
	Microsomal fraction	−	−	0.07 ± 0.02^a^
		+	−	1.29 ± 0.16^a^
		+	+	2.66 ± 0.50^a^

Errors represent the standard deviation of triplicate analysis.

^a^Total protein yields were estimated from the eYFP fluorescence of the suppression product in relation to the calculated total yields and eYFP fluorescence of the wild type protein synthesized under identical conditions.
