# Supplementary material for: Cell-free synthesis of functional human epidermal growth factor receptor: Investigation of ligand-independent dimerization in Sf21 microsomal membranes using non-canonical amino acids
Source: Sci Rep. 2016 Sep 27;6:34048. doi: 10.1038/srep34048 (PMC5037433; doi:10.1038/srep34048)
Supplement: Supplementary Information [file srep34048-s1.pdf]

Supplementary Information

Cell-free synthesis of functional human epidermal growth factor receptor: Investigation of ligand-independent dimerization in *Sf*21 microsomal membranes using non-canonical amino acids

Robert B. Quast[1], Biljana Ballion[2], Marlitt Stech[1], Andrei Sonnabend[1], Balázs R. Varga[3], Doreen A. Wüstenhagen[1], Péter Kele[3], Stefan Schiller[2], Stefan Kubick[1]

[1]Fraunhofer Institute for Cell Therapy and Immunology (IZI), Branch Bioanalytics and Bioprocesses (IZI-BB), Am Mühlenberg 13, D-14476 Potsdam, Germany

[2]Institute for Macromolecular Chemistry, University of Freiburg, Stefan-Meier-Str. 31, D-79104 Freiburg, Germany & Freiburg Institute for Advanced Studies (FRIAS), School of Soft Matter Research,University of Freiburg, Albertstr. 19, D-79104 Freiburg, Germany & Center for Biosystems Analysis (ZBSA), University of Freiburg, Habsburger Str. 49, D-79104 Freiburg, Germany

[3]Chemical Biology Research Group, Hungarian Academy of Sciences, CNS, IOC, Magyar tudósok krt. 2, H-1117 Budapest, Hungary

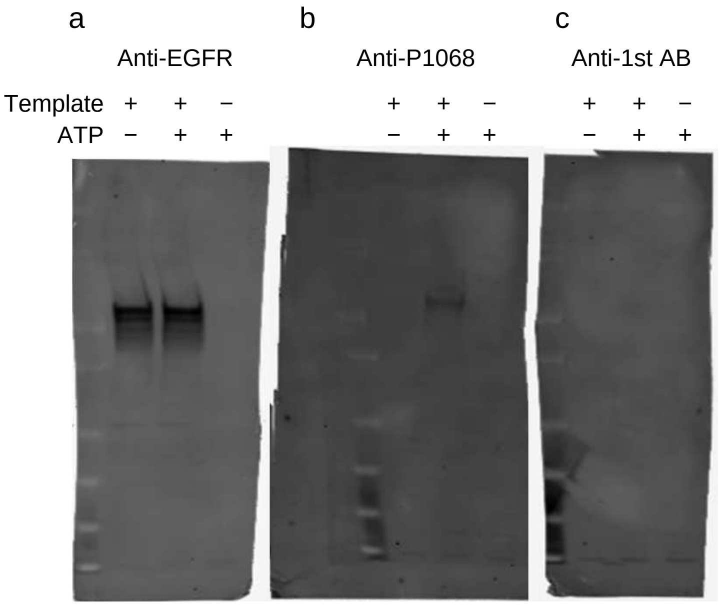

Supplementary figure 1 | Original unmodified western blots shown in figure 1e. a) Total EGFR, b) EGFR phosphorylated at tyrosine 1068 and c) application of secondary HRP-linked antibody only.

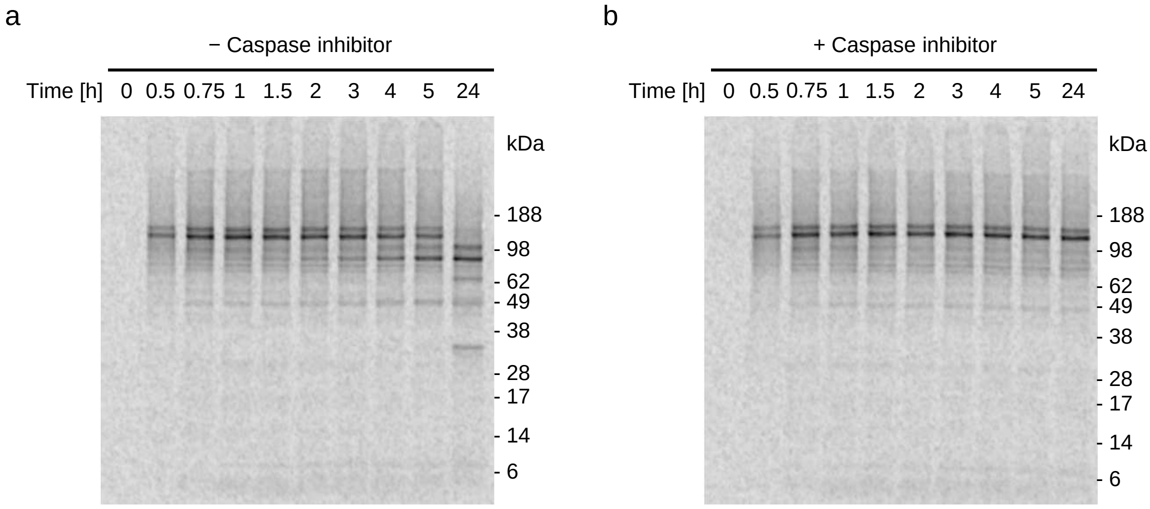

Supplementary figure 2 | Influence of caspase inhibitor on integrity of cell-free synthesized EGFR-eYFP. IRES-mediated synthesis of EGFR-eYFP in the presence of poly G was performed for 24 hours with (a) and without the caspase inhibitor Z-VAD-FMK (b). Samples were taken at the indicated time points and separated by electrophoresis followed by autoradiography. Isotopic labeling was achieved by ^{14}C-leucine supplementation.

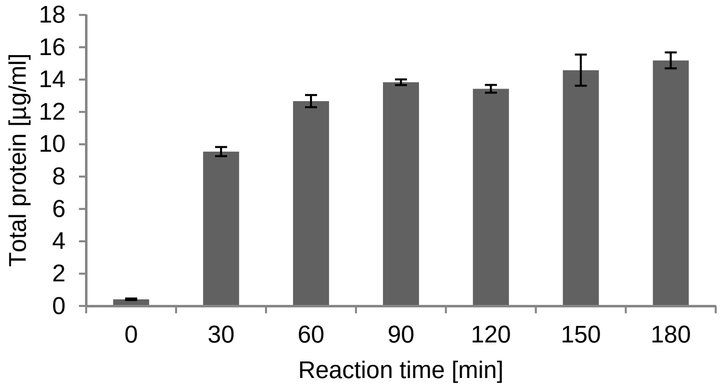

Supplementary figure 3 | Reaction lifetime of IRES-mediated synthesis in the presence of poly G. The synthesis of EGFR-eYFP was performed for 180 minutes. Samples were taken at the indicated time points, total protein was precipitated by hot TCA precipitation and analyzed by β-scintillation counting. Isotopic labeling was achieved by ^{14}C-leucine supplementation.

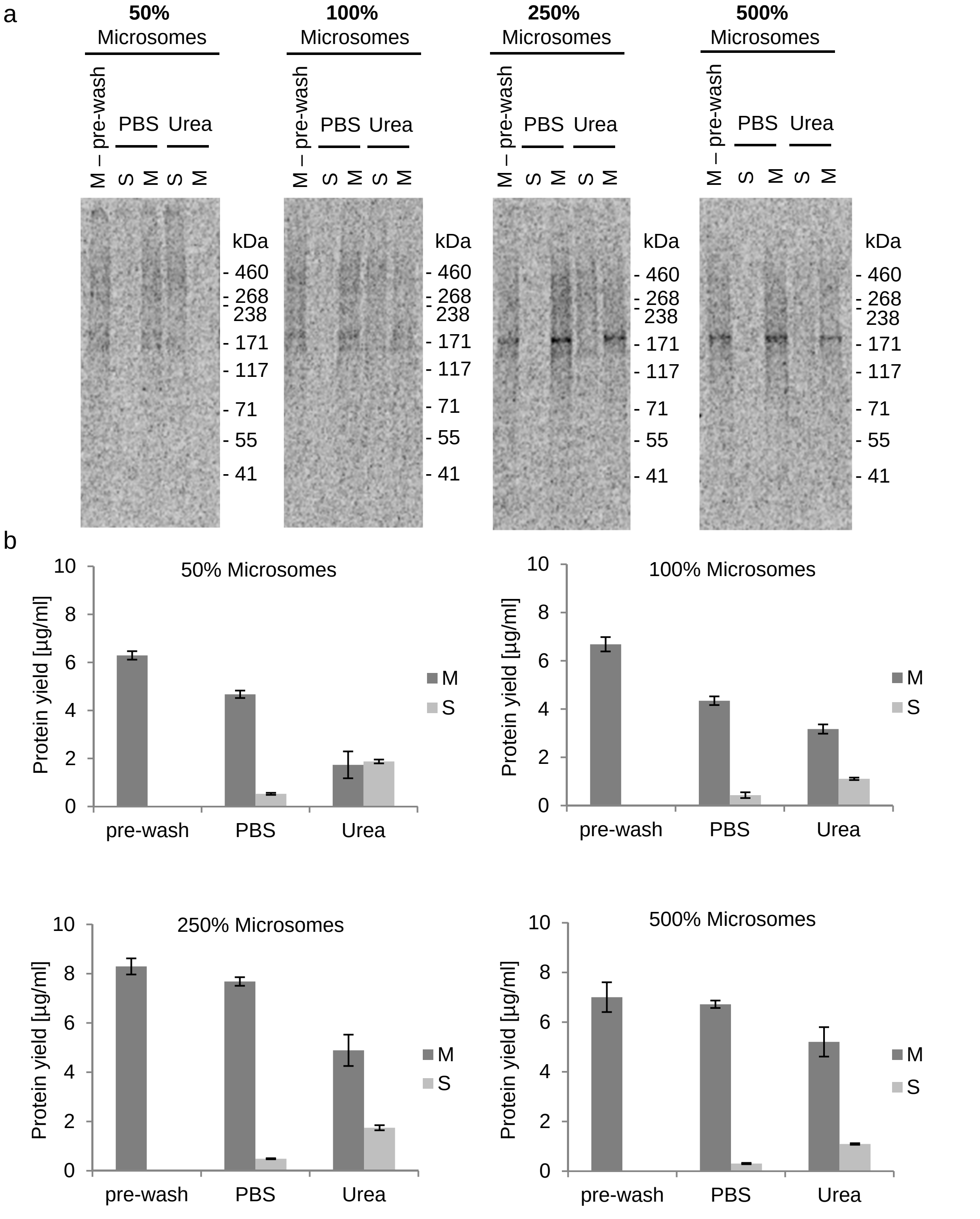

Supplementary figure 4 | Analysis of the amount of membrane-integrated receptors by autoradiography (a) and β-scintillation counting (b) after washing the microsomes with urea. IRES-mediated cell-free synthesis of EGFR-eYFP was performed in the presence of different amounts of microsomes, whereas 100% represents the standard microsome amount used during this study. Isotopic labeling was achieved by ^{14}C-leucine supplementation. Microsomal fractions of the different cell-free reactions containing 50%, 100%, 250% and 500% microsomes were either washed with PBS or urea (4.5 M urea in PBS). The washing step was performed by pipetting the microsomes up and down until a homogenous solution was obtained followed by incubation on ice for 30 min. Samples were subsequently centrifuged and the resulting supernatant (S) and washed microsomal fraction (M) were analyzed by autoradiography and β-scintillation counting. M pre-wash: Analysis of microsomal fraction before the washing step. Error bars represent the standard deviation of triplicate analysis.

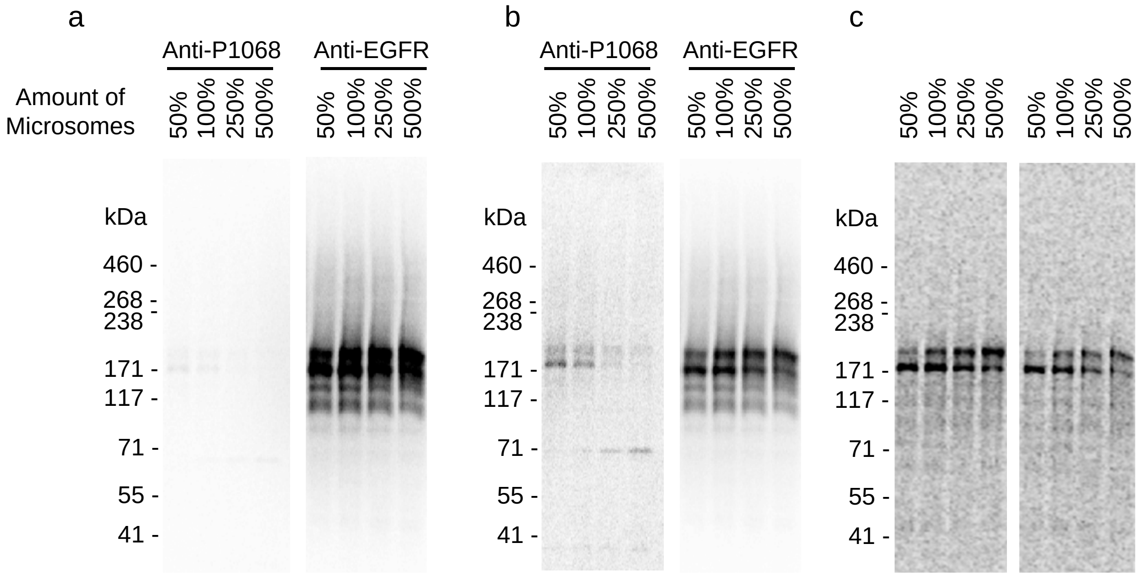

Supplementary figure 5 | Western Blot (a and b) and autoradiograph (c) of microsomal fractions with EGFR-eYFP after incubation in kinase buffer. IRES-mediated cell-free synthesis of EGFR-eYFP was performed in presence of different amounts of microsomes, whereas 100% represents the standard microsome amount used during this study. Isotopic labeling was achieved by ^{14}C-leucine supplementation. Western blot membranes were blocked in TBS buffer containing 2% BSA and 0.1% Tween-20 for 1 hour and subsequently incubated with “EGF Receptor (D38B1) XP® Rabbit mAb 4267” or “Phospho-EGF Receptor (Tyr1068) (D7A5) XP® Rabbit mAb 3777” primary antibodies diluted 1:1000 over night at 4°C. “Anti-rabbit IgG, HRP-linked Antibody 7074” diluted 1:2000 was used as a secondary antibody and detection was carried out using the “Amersham ECL Select Western Blotting Detection Reagent” (GE Healthcare) and the “Typhoon Trio+ Variable Mode Imager” (GE Healthcare). Western blot membranes shown in (a) were scanned simultaneously, while the membranes depicted in (b) were scanned one after another and have been adapted in contrast and brightness for better visibility of faint bands. The autoradiograph shown in (c) corresponds to the western blot membrane depicted in (a) and (b).

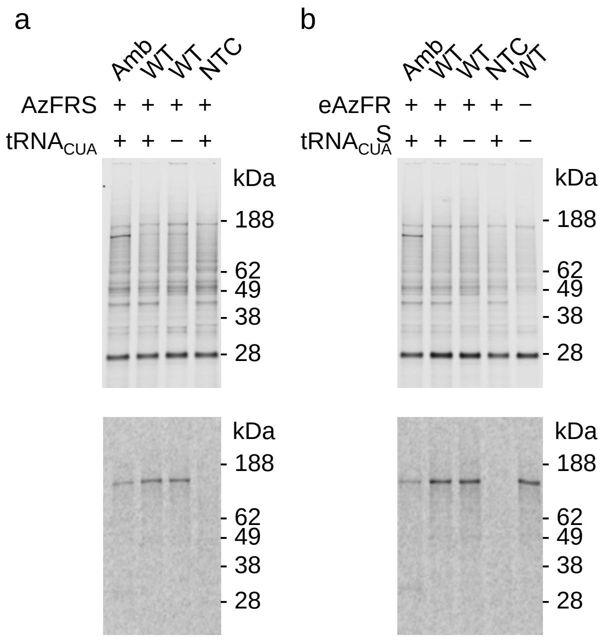

Supplementary figure 6 | Specificity of AzF incorporation by different *E. coli* tyrosyl-tRNA synthetase mutants. Cell-free reactions were performed using the standard EGFR-eYFP template without (WT) and with amber codon at position 687 (Amb) under standard conditions in the presence of AzF. The synthetases AzFRS (Thr37, Ser182, Ala183; a) and eAzFRS (Thr37, Ser182, Ala183, Arg265; b) and $tRNA_{CUA}$ were supplemented as indicated. In-gel fluorescence (top) and autoradiography (bottom) of microsomal fractions after treatment with DyLight650 phosphine. Isotopic labeling was achieved by ^{14}C-leucine supplementation. NTC: control reaction without DNA template.

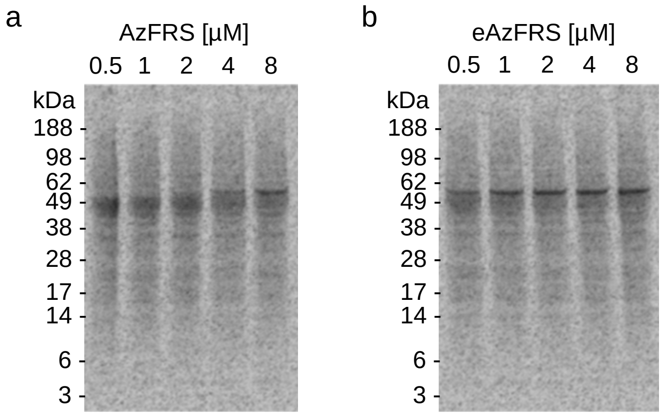

Supplementary figure 7 | Efficiency of AzF incorporation by different *E. coli* tyrosyl-tRNA synthetase mutants. Cell-free reactions were performed under standard conditions using a firefly luciferase template with amber codon at position 21 in the presence of AzF, $tRNA_{CUA}$ and AzFRS (a) or eAzFRS (b) at the given concentrations and analyzed by autoradiography after electrophoretic separation. Isotopic labeling was achieved by ^{14}C-leucine supplementation.

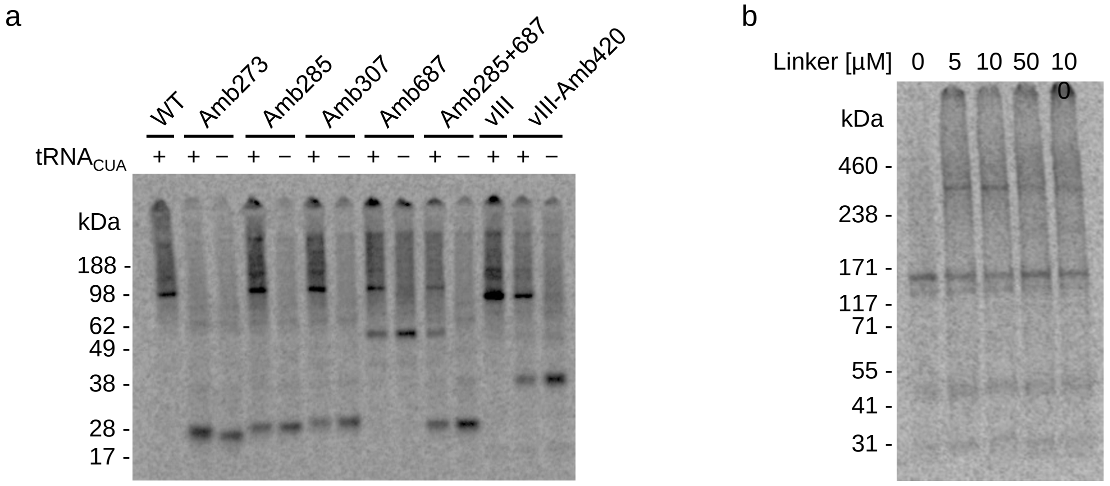

Supplementary figure 8 | Suppression efficiency of different amber variants synthesized in the orthogonal cell-free translation system and concentration dependency of strain-promoted cycloaddition with bis-COMBO linker. a) Autoradiography of cell-free synthesis reactions using wild type EGFR-eYFP and vIII templates including CrPV-IRES and corresponding amber variants (Amb) in the presence (+) or absence of tRNA$_{CUA}$ (−). b) Autoradiography of cell-free synthesized vIII deletion mutant with incorporated AzF at position 420 after treatment with different concentrations of bis-COMBO linker for 2 h at RT in kinase buffer. Isotopic labeling was achieved by ^{14}C-leucine supplementation.

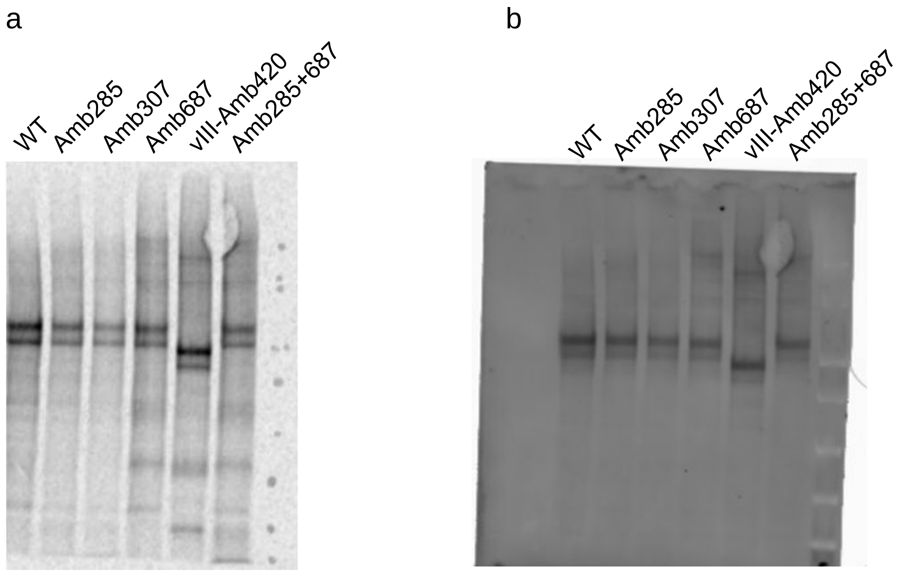

Supplemetary figure 9 | Original unmodified autoradiogram (a) and western blot (b) shown in figure 4b and c, respectively.

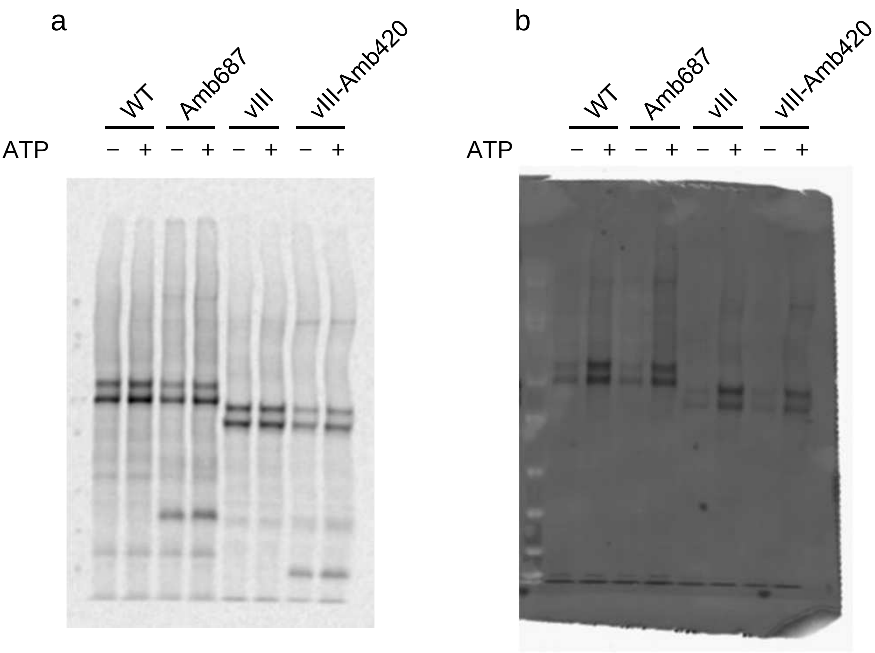

Supplemetary figure 10 | Original unmodified autoradiogram (a) and western blot (b) shown in figure 5b and c, respectively.

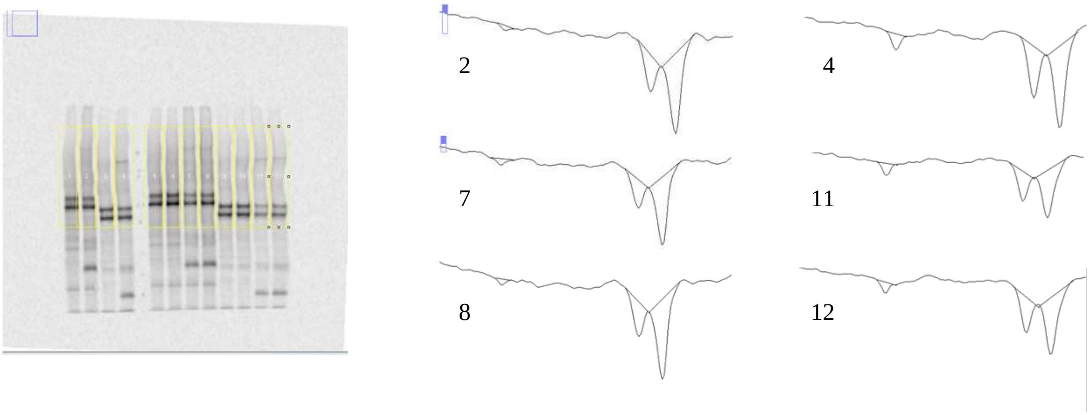

	Area
1	165.092
2	1552.225
3	3697.368
4	552.406
5	2160.154
6	3735.468
7	230.092
8	1164.326
9	3006.711
10	172.971
11	1397.740
12	3531.782
13	387.335
14	1149.083
15	2321.933
16	330.042
17	1375.790
18	2679.882
19	154688.633
20	161109.169

Construct	Lane	Band	Area	sum	ratio	%	mean	SD
EGFR-eYFP-AzF687	2	Cross-linked	165.092				4.06	1.28
		glycosylated	1552.225					
		nonglycosylated	3697.368	5249.593	0.031448533	3.14		
	7	Cross-linked	230.092					
		glycosylated	1164.326					
		nonglycosylated	3006.711	4171.037	0.055164219	5.52		
	8	Cross-linked	172.971					
		glycosylated	1397.74					
		nonglycosylated	3531.782	4929.522	0.035088798	3.51		
vIII-AzF420	4	Cross-linked	552.406				9.56	1.52
		glycosylated	2160.154					
		nonglycosylated	3735.468	5895.622	0.093697662	9.37		
	11	Cross-linked	387.335					
		glycosylated	1149.083					
		nonglycosylated	2321.933	3471.016	0.111591246	11.16		
	12	Cross-linked	330.042					
		glycosylated	1375.79					
		nonglycosylated	2679.882	4055.672	0.081377883	8.14		

Supplementary figure 11 | Amount of cross-linked receptor molecules using the bis-COMBO-linker. Quantification was carried out using the ImageJ software.

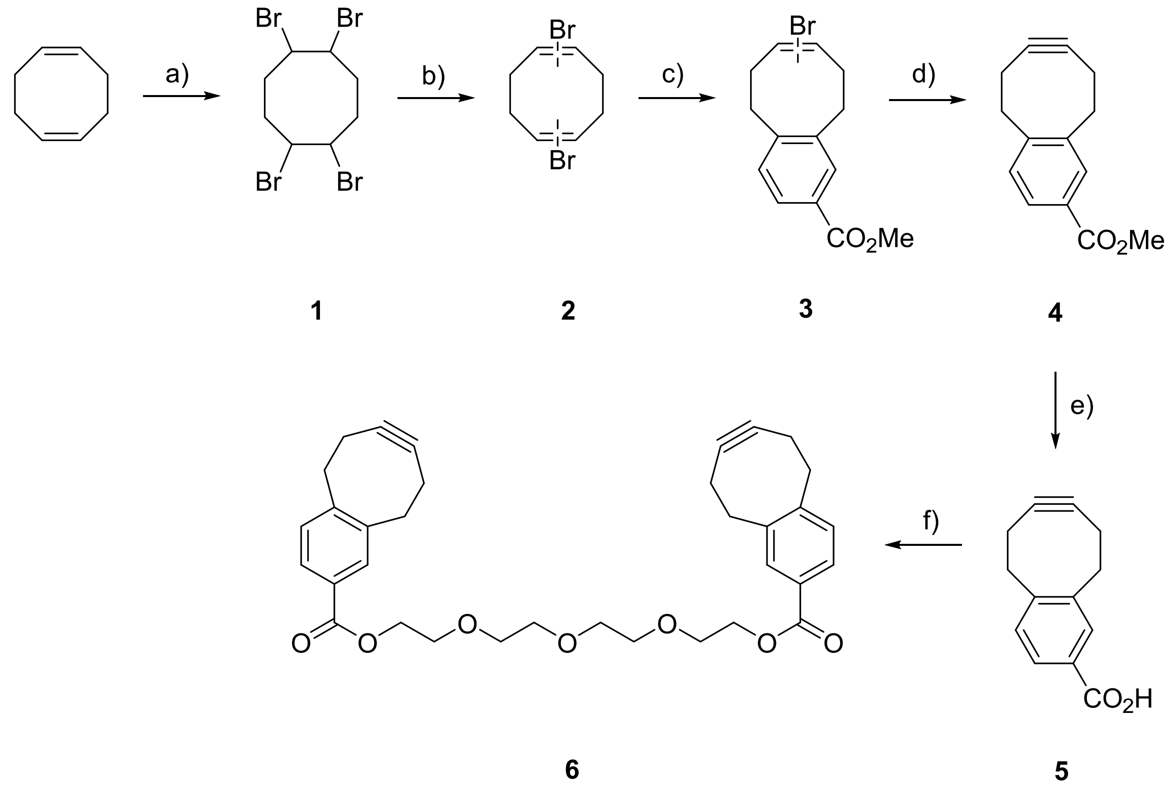

Supplementary figure 12 | Synthesis of COMBO-tetraethylene glycol-COMBO linker. a) 1,5-cis-cyclooctadien (1.0 eq), Br2 (4.6 eq), CH2Cl2, -78°C → RT, 1 h, 63% b) 1 (1.0 eq), KOtBu (2.4 eq), Et2O, -78°C → RT, overnight, 81% c) 2 (1.0 eq), KOtBu (6.5 eq), [18]-crown-6 ether, hexane, 1.5 h, Methyl coumalate (0.36 eq), overnight, 25% d) 3 (1.0 eq), KOtBu (2.5 eq), [18]-crown-6 ether, hexane, 1.5 h, 58-60°C, 29% e) 4 (1.0 eq), LiOH (20 eq), dioxane, 2 h, 60% f) 5 (2.5 eq), TEG (1.0 eq), EDC, DMAP, CH2Cl2, 0°C → RT, overnight, 36%.
